# The Endometriotic Tumor Microenvironment in Ovarian Cancer

**DOI:** 10.3390/cancers10080261

**Published:** 2018-08-07

**Authors:** Jillian R. Hufgard Wendel, Xiyin Wang, Shannon M. Hawkins

**Affiliations:** Department of Obstetrics and Gynecology, Indiana University School of Medicine, Indianapolis, IN 46202, USA; jhufgard@iu.edu (J.R.H.W.); xw49@iu.edu (X.W.)

**Keywords:** ovarian cancer, endometriosis, tumor microenvironment, miRNA molecules, genes, hypoxia, inflammation, model systems

## Abstract

Women with endometriosis are at increased risk of developing ovarian cancer, specifically ovarian endometrioid, low-grade serous, and clear-cell adenocarcinoma. An important clinical caveat to the association of endometriosis with ovarian cancer is the improved prognosis for women with endometriosis at time of ovarian cancer staging. Whether endometriosis-associated ovarian cancers develop from the molecular transformation of endometriosis or develop because of the endometriotic tumor microenvironment remain unknown. Additionally, how the presence of endometriosis improves prognosis is also undefined, but likely relies on the endometriotic microenvironment. The unique tumor microenvironment of endometriosis is composed of epithelial, stromal, and immune cells, which adapt to survive in hypoxic conditions with high levels of iron, estrogen, and inflammatory cytokines and chemokines. Understanding the unique molecular features of the endometriotic tumor microenvironment may lead to impactful precision therapies and/or modalities for prevention. A challenge to this important study is the rarity of well-characterized clinical samples and the limited model systems. In this review, we will describe the unique molecular features of endometriosis-associated ovarian cancers, the endometriotic tumor microenvironment, and available model systems for endometriosis-associated ovarian cancers. Continued research on these unique ovarian cancers may lead to improved prevention and treatment options.

## 1. Introduction

Endometriosis is a debilitating disease that is estimated to affect up to 5 million U.S. women and girls. Endometriosis results in considerable morbidity, including pelvic pain, multiple operations, infertility, and negative effects on psychosocial quality of life [1,2,3,4,5]. Unfortunately, endometriosis is also a significant risk factor for development of ovarian cancer [6]. The presence of endometriosis increases the risk of ovarian endometrioid, low-grade serous, and clear-cell adenocarcinoma by up to 8.9-fold but not high-grade serous adenocarcinoma [7,8,9,10,11,12]. Thus, ovarian endometrioid, low-grade serous, and clear-cell adenocarcinomas are considered endometriosis-associated ovarian cancers. Ovarian cancer is considered a top-five cancer killer in U.S. women, claiming more than 14,000 lives in 2015 [13]. Therefore, 5 million U.S. women and girls with endometriosis are at risk for developing deadly ovarian cancer. Fortunately, ovarian endometrioid and clear-cell adenocarcinoma represent roughly 20% of all ovarian cancers and account for less than 10% of deaths [14,15,16]. Clinically, studies suggest that co-occurrence of endometriosis with ovarian cancer is associated with an improved prognosis [17,18,19,20]. Important factors in this improved prognosis include discovery at early age and early stage disease in women with endometriosis at time of ovarian cancer staging [21,22,23,24], but may also represent the unique biology from the endometriotic tumor microenvironment. This review will focus on the contributions of the endometriotic tumor microenvironment to ovarian cancer biology.

## 2. Unique Molecular Features of Endometriosis-Associated Ovarian Cancer

Each histotype of epithelial ovarian cancer is thought to arise from a distinct precursor lesion. For example, endometriosis is thought to give rise to both ovarian endometrioid and clear-cell adenocarcinomas [25]. Recently, sophisticated proteomic tracing studies suggest that ovarian endometrioid adenocarcinomas arise from secretory cells of endometriosis or the endometrium, while ovarian clear-cell adenocarcinomas arise from ciliated cells. Importantly, it is hypothesized that the unique cellular environment dictates the development of ciliated or secretory cells, which then gain mutations to become malignant [26]. Recently, next-generation sequencing studies showed mutations in cancer-driver genes (i.e., AT-rich interaction domain 1A (*ARID1A*), Phosphatidylinositol-4, 5-bisphosphate 3-kinase catalytic subunit alpha (*PIK3CA*), and Kirsten rat sarcoma viral oncogene homolog (*KRAS*)) in deep infiltrating endometriotic lesions, supporting the idea that the endometriotic microenvironment facilitates mutations [27]. Because deep infiltrating endometriotic lesions do not pose a risk of malignant transformation, the unique contributions of driver mutations in these particular endometriotic lesions are still relatively unknown [27]. Interestingly, these mutations in cancer-driver genes were only present in glandular epithelium and not underlying stroma [27]. These data support the idea that both epithelium and stromal populations of deep infiltrating endometriosis do not represent similar clonal populations. Further, this data may represent the idea that unique stromal populations are recruited to the area [28,29]. Detailed studies of unique genetic contributions of both epithelial and/or stromal compartments in malignant transformation are needed.

Studies examining endometriotic lesions and ovarian cancer from the same patient have shown concordant mutations in *ARID1A*, phosphatase and tensin homolog (*PTEN*), *PIK3CA*, and *KRAS*, suggesting that mutations in endometriosis cause a predisposition to ovarian cancer [30,31,32,33]. Mutations in *KRAS* and *ARID1A* have been discovered in endometriosis, including ovarian endometriosis and deep infiltrating endometriosis [27,34]. Loss of ARID1A is higher in atypical endometriosis and non-atypical endometriosis adjacent to ovarian cancer than non-atypical endometriotic distal lesions [30,32,35,36,37,38,39]. In general, both endometrioid and clear cell ovarian cancer with or without endometriosis have common high frequency mutations in *ARID1A*, *PIK3CA*, catenin betat 1 (*CTNNB1*), *PTEN*, and *KRAS* [33,40,41,42,43,44,45]. In terms of unique molecular features, 29% of low-grade ovarian endometrioid adenocarcinomas with concurrent endometriosis contained mutations in *KRAS* compared to 3% of low-grade endometrioid adenocarcinomas lacking endometriosis [33]. Importantly, Ishikawa et al. showed high frequency of *ARID1A* mutations and one patient with both *ARID1A* and *KRAS* mutations in endometriosis-associated ovarian cancers [43]. The contributions of both ARID1A and KRAS warrant further study in terms of endometriosis, the endometriotic tumor microenvironment, and endometriosis-associated ovarian cancer.

In terms of low-grade serous tumors, an A to T substitution in *BRAF* has been identified in 36–68% of low-grade serous ovarian cancers and is associated with improved prognosis [46,47,48]. Additionally, increased expression of B-raf proto-oncogene, serine/threonine kinase (BRAF) was also noted in eutopic and ectopic endometrium of women with endometriosis when compared to control endometrium [49]. The contributions of BRAF to endometriosis and endometriosis-associated ovarian cancers, specifically, low-grade serous ovarian cancers are understudied.

In addition to mutational changes, epigenetic changes play a role in both endometriosis and endometriosis-associated ovarian cancers. Methylation changes in both endometriosis and endometriosis-associated ovarian cancer have recently been reviewed [50,51]. Along those lines, endometriosis tissues have decrease expression of ten-eleven translocation genes (*TET1*, *TET2*, and *TET3*), which convert 5-methylcytosine to 5-hydroxymethlcytosine and play a role in changes in levels of 5-hydroxymethylcytosine marks in endometriosis tissues and blood [52]. Unfortunately, the authors did not assess 5-hydroxymethlcytosine marks in specific genes. Further studies are needed in endometriosis-associated ovarian cancer to examine changes in these and other alternative DNA marks. MicroRNA (miRNA) molecules, which are also considered epigenetic changes, are dysregulated in endometriosis (reviewed in [53]). While dysregulated miRNAs in epithelial ovarian cancers have been recently reviewed [54,55], dysregulated miRNA molecules in endometriosis-associated ovarian cancers have not been individually reviewed. Given that miRNA molecules can be secreted from cells, we have included miRNA molecules under endometriotic tumor microenvironment (below).

A challenge to studies on the endometriotic tumor microenvironment is the rarity of clinical samples of ovarian cancer with concurrent endometriosis and the rigor of details provided for patient characterization. Given over 22,000 women will be diagnosed with ovarian cancer in 2016 [13], only 10% will be endometrioid and roughly 10% will be clear-cell [14,15,16]. Additionally, a majority of women with endometriosis-associated ovarian cancers do not have endometriosis at time of staging. Roughly 30% of ovarian endometrioid or clear-cell adenocarcinomas will have concurrent endometriosis, further narrowing the number of tumors to study with concurrent endometriosis [56,57,58,59]. Many studies do not describe the patient population in terms of absence or presence of endometriosis, leaving readers to believe that the women may not have endometriosis, which may not be accurate. Efforts for data harmonization for rare tumors may improve reproducibility. Using well-characterized samples, Banz et al. used transcriptome microarray analysis to evaluate normal ovary, endometriomas, and endometrioid ovarian cancer with and without endometriosis [60]. The results showed a small group of cytokines dysregulated in ovarian cancers with endometriosis, consistent with the inflammatory milieu of endometriosis [60]. Additionally, Zhang et al. showed a unique gene signature in ovarian endometrioid adenocarcinoma with concurrent endometriosis compared to ovarian endometrioid adenocarcinoma without concurrent endometriosis [61]. Highly dysregulated signaling pathways included nuclear factor kappa B (NFkB), transforming growth factor beta (TGFβ), and KRAS signaling [61]. Most likely there are contributions from genetics and epigenetics that may be mediated from the endometriotic tumor microenvironment [62]. However, further studies are needed to examine how endometriosis affects ovarian cancer.

## 3. The Unique Endometriotic Tumor Microenvironment

**Figure 1:**
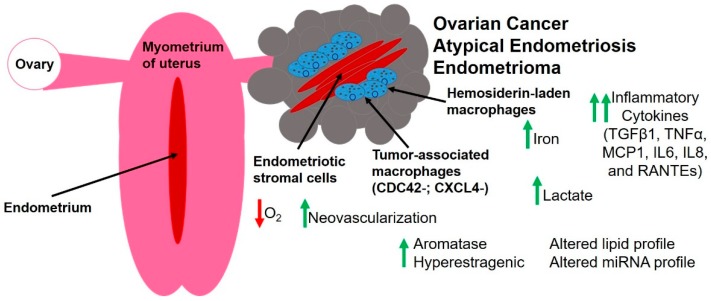
Composition of the endometriotic tumor microenvironment. Endometriosis represents a pathologically benign disease. Endometriosis may be classified into endometriomas, superficial peritoneal disease, or deep infiltrating endometriosis (invasion > 5 mm). Although deep infiltrating endometriosis is invading, typically into the muscularis layer of the bowel, it is clinically not associated with ovarian cancer. Endometriomas are epithelial lined cysts of the ovary, which can be filled with a brown cyst fluid, and thus the name “chocolate cysts.” Endometriomas can be associated with ovarian cancer, with atypical endometriomas having a higher risk of malignant transformation. Atypical endometriomas are characterized by epithelial cells with enlarged hyperchromatic and pleomorphic nuclei, with cellular crowding and high nuclear-to-cytoplasmic ratio. The altered endometriotic tumor microenvironment may lead to malignant transformation or propagation of proliferative potential [107]. RANTES: regulated on activation normal T cell expressed and secreted; MCP1: monocyte chemotactic protein-1; IL: interleukin; TGFβ1: transforming growth factor beta 1; TNFα: tumor necrosis factor alpha; CDC42: cell division cycle 42; CXCL4: chemokine (C-X-C motif) ligand 4.

While the pathogenesis of endometriosis is still largely poorly understood, the most accepted theory is the implantation theory following retrograde menstruation (reviewed in [63]). Most menstruating women have retrograde menstruation [64], but only 10% have endometriosis [1,2,3], suggesting that unique conditions occur in women with endometriosis. The endometriotic microenvironment contains multiple cell types—endometrial epithelial cells, stromal fibroblasts, endothelial cells, and immune cells—as well as inflammatory mediators, metabolic waste products such as iron from the breakdown of red blood cells, steroid hormones, and small RNA molecules. Thus, it is not surprising that the conditions found in endometriosis are also advantageous to the growth and development of ovarian cancer. However, very little is known about how these stressful conditions directly affect ovarian cancer. In this section, we will describe these important factors within the scope of endometriosis and how these important factors pertain to ovarian cancer. Figure 1 summarizes graphically key players in the endometriotic tumor microenvironment as it pertains to ovarian cancer.

### 3.1. Hypoxia and Endothelial Cells

Hypoxia is thought to be critical to the survival and invasion of endometriotic cells through multiple mechanisms including autophagy [65,66,67,68], TGFβ signaling [69], and signal transducer and activator of transcription 3 (STAT3) signaling [70,71,72]. In endometriosis, hypoxia stabilizes hypoxia inducible factor-1α (HIF1A) which downregulates dual-specificity phosphatase-2 (DUSP2) directly and indirectly through miR-20a [73]. Ultimately, this downregulation leads to increased angiogenesis and proliferation through activation of extracellular signal-regulated kinase (ERK) signaling cascades [73,74]. As such, molecular immunohistochemistry shows a high correlation between precursor endometriosis lesions and matched clear-cell adenocarcinomas for expression of HIF1A and phosphorylated mechanistic target of rapamycin kinase (P-mTOR) [75]. Importantly, vascular endothelial growth factor (VEGF), leptin (LEP), cysteine rich angiongenic inducer 61 (CYR61), and osteopontin (SPP1) work together in response to hypoxia to establish a local vascular network within the endometriotic lesion [74]. In addition to neoangiogenesis mediated through HIF1A, as endometriotic lesions undergo hypoxia and inflammation from repeated menstrual cycles, the expression of tissue factor increases. Tissue factor is a critical protein for extrinsic coagulation cascade, leading to hypercoagulation. Clinically, women with clear-cell ovarian cancer have more frequent venous thromboembolism [76]. Hypoxia may also lead to cellular proliferation through estrogen receptor, leptin, and prostaglandin modulation [77]. These studies suggest that the hypoxic microenvironment of endometriosis plays a role in not only the potentiation of endometriosis by promoting cell proliferation and nutrient availability through vascularization but may also play roles in outcomes for women with clear-cell ovarian cancer. The increased expression of HIF1A in endometriosis may represent a novel therapeutic target for endometriosis or ovarian cancer [78].

### 3.2. Fibroblasts and Extracellular Matrix Components

Endometriosis is pathologically complex, containing endometrial epithelial and stromal fibroblasts outside the uterine cavity, alongside invading hemosiderin-laden macrophages [79]. The endometriotic extracellular matrix (ECM) plays a significant role in paracrine/autocrine signaling between epithelial and stromal cells [80,81,82,83]. Studies have shown unique functional properties of primary cultures of human endometrial stromal fibroblasts from women with endometriosis compared to cultures from women without endometriosis. Specifically, fibroblast cultures from women with endometriosis have a deficiency in decidualization, the differentiation process by which the uterus prepares for pregnancy [84]. Additionally, these fibroblasts from women with endometriosis have increased ERK signaling, high proliferative potential from progesterone resistance, and acquire an inflammatory phenotype [85,86,87,88,89]. While the importance of stromal-epithelial crosstalk is noted in embryo implantation in the uterus [80], the role of similar crosstalk in endometriosis or epithelial ovarian cancers is still understudied but may represent a key component of the endometriotic tumor microenvironment.

To examine the tumor microenvironment in ovarian cancer, Zhang et al. used computer-aided image analysis and showed that the number of cancer-associated fibroblasts, as indicated by cells positive for smooth muscle antigen, was higher in epithelial ovarian cancers compared to benign adnexal masses. Unfortunately, the specific histology of ovarian cancers and the pathology of the benign adnexal masses were not described in these studies. Large numbers of similarly staining cancer-associated fibroblasts were also found in omental metastatic lesions [90]. Co-culture of cancer-associated fibroblast with ovarian cancer cell lines (SKOV3, CAOV3) led to increased invasion and migration when compared to ovarian cancer cell lines grown in co-culture with normal fibroblasts [90]. One of the main questions regarding cancer-associated fibroblasts is how and why they are becoming activated to benefit tumor cells. Mitra et al. proposed that ovarian cancer cells reprogram fibroblasts into cancer-associated fibroblasts through miRNA expression changes [91]. Specifically, cancer-associated fibroblasts have a significant downregulation of miR-31 and miR-214 and upregulation of miR-155. C-C motif ligand 5 (CCL5), a chemokine known to be highly upregulated in ovarian cancers, is a direct target of miR-214. Similarly, endometriomas have high expression of chemokines and dysregulated miRNA expression [92]. Advancements in the understanding of the role of non-epithelial ovarian cancer cells in ovarian cancer may lead to better treatments which block tumor promotion brought on by tumor adjacent cells.

### 3.3. Immune Cells and Inflammatory Mediators

Dysregulated inflammation plays a key role in endometriosis-associated pathology [93]. For example, Capobianco and Rovere-Querini provide an in-depth review of the role of macrophages in endometriosis, showing a relationship between components of the endometriotic microenvironment such as high iron, hypoxia, and angiogenesis with macrophage recruitment and activation [94]. Additionally, a syngeneic mouse model of endometriosis showed that endometriotic lesions failed to grow without macrophages, and if macrophages were removed after implantation, angiogenesis was halted, blocking the progression of the endometriotic lesion [95]. Further, Canet et al. suggest that retainment of a specific macrophage population in endometriomas, the cell division cycle 42 (CDC42)-positive population, protects endometriomas from malignant transformation [96]. Similarly, platelet factor 4 (PF4) also known as chemokine (C-X-C Motif) ligand 4 (CXCL4) is highly expressed on macrophages in endometriomas, but not on tumor-associated macrophages of clear cell ovarian cancers [97]. Thus, specific details of the macrophage population in endometriosis and ovarian cancer are important and require further study.

Transcriptomic work on endometriomas showed that the inflammatory cytokine transforming growth factor beta 1 (TGFβ1), regulates other inflammatory mediators relevant to endometriosis, including tumor necrosis factor alpha (TNFα) and interleukin-6 (IL6) [92]. These inflammatory mediators are highly elevated in peritoneal fluid from women with endometriosis [98,99,100,101]. The acute and chronic inflammation of endometriosis is a response to the invading tissue, leading to the release of regulated on activation normal T cell expressed and secreted (RANTES), monocyte chemotactic protein-1 (MCP1), and interleukin-8 (IL8), which act as chemoattractants recruiting more macrophages to the area [102]. In terms of the endometriotic tumor microenvironment, the promotion of tumor invasion via macrophages may be dependent on TNFα [103], which is elevated in women with endometriosis [98,99]. Along the same lines, work using an estrogen receptor beta (ERβ)-overexpressing syngeneic mouse model of endometriosis suggests that non-genomic effects of ERβ play a role in the TNFα-mediated dysregulation of endometriosis progression [104]. Encouragingly, treatment of a syngeneic mouse model of endometriosis with a long-acting TNFα-blocking agent decreased endometriotic implant size [105]. However, treatment of women with rectovaginal nodules with infliximab, a TNFα monoclonal antibody, had no improved clinical effect over placebo [106]. Understanding the immune response to misplaced endometrial tissue will be a large factor in understanding the onset and progression of endometriosis and lead to a better understanding of how endometriosis creates a unique and potentially tumor-promoting microenvironment.

### 3.4. Altered Metabolism

Endometriotic cysts contain blood. When blood is metabolized, heme and iron are released into the microenvironment [107]. Because of this, endometriotic cysts contain higher iron levels than other benign ovarian cysts [108]. Consequently, an iron-rich microenvironment can lead to increased proliferation, DNA synthesis, and adhesion, and promote chronic inflammation, allowing for the spread of endometriosis [107]. High iron also leads to excessive oxidative stress, which creates a microenvironment conducive to the induction of mutations and has been linked to cancer development in the liver and lung [107,109]. Shigetomi et al. outlines how endometriotic cells under oxidative stress from excess iron are able to bypass cell cycle checkpoints after DNA damage by overexpressing hepatocyte nuclear factor-1 beta (HNF1B), which activates forkhead box transcription factors and alters miRNA expression promoting cell survival [110]. Due to the excess iron exposure, endometriotic cysts have higher expression of lactose dehydogenase, lipid peroxidase, and 8-hydroxy-2′-deoxyguanosine. High expression of these markers of oxidative stress link endometriosis, high iron, and higher frequencies of gene mutations [108]. These data corroborate the hypothesis that endometriosis produces a high iron microenvironment that may lead to increased DNA damage through oxidative stress, but also promotes cell survival, leading to a highly mutated subpopulation of cells that continue to grow [111].

Alongside high iron levels, endometriotic peritoneal fluid has elevated lactate. Further, endometriotic lesions express high levels of glycolysis genes compared to eutopic endometrium [112]. Increased expression of HNF1α in the endometriotic peritoneum leads to the conversion of glucose to lactate in a process known as the “Warburg Effect,” known for its promotion of cell survival in stressful microenvironments [113]. Lipidomics has also been pursued for understanding the metabolomic profile of the endometriotic microenvironment. Lipid profiling studies on endometrial aspirates have shown a reduction of saturated diacylglycerols and triacylglycerols in endometriosis patients compared to healthy controls [114]. In fact, this study generated a panel of 123 metabolites which were differentially expressed in endometriosis women and correctly identified 86% of samples to either the endometriosis or control group [114]. A similar study on endometrial biopsies used five lipid metabolites as biomarkers and were able to predict endometriosis with 75% specificity and 90.5% sensitivity [115]. A true model of the endometriotic tumor microenvironment should include increased iron levels, higher levels of glycolysis-associated proteins, and endometriosis-associated lipidomic profiles.

### 3.5. Steroid Hormones

Endometriosis is an estrogen-responsive disorder with lesion-level hyperestrogenism. Specifically, endometriotic tissue differs from eutopic endometrial tissue by the high expression of aromatase (CYP19A1) and 17β-hydroxysteroid-dehydrogenase (17β-HSD) type 1 and the absence of 17β-HSD type 2 [107,116]. Aromatase converts androstenedione or testosterone to estrone and estradiol at the level of the endometriotic microenvironment. High levels of estradiol have been linked to IL8 and RANTES production, which facilitate proliferation, inflammation, and feedback to increased expression of aromatase [107,117]. Aromatase activity is also stimulated through prostaglandin E_2_, an inflammatory product of cyclooxygenase 1 and 2 (COX1/2), found in endometriotic lesions in high levels [118]. Inhibitors of prostaglandin E2 receptor show promising effects in a xenograft model of endometriosis [119]. At the endometriotic lesion level, there is significant feed forward production and maintenance of estrogen, associated with pro-tumorigenic qualities. Medical management of endometriosis with oral contraceptives lowers overall steroid hormone levels. This may explain why the protection from combined oral contraceptive therapy on ovarian cancer risk is more robust for women with endometriosis (odds ratio 0.21 (0.08–0.58), *p* = 0.003) compared to non-endometriosis population (odds ratio 0.47 (0.37–0.61, *p* < 0.001)) [120]. Thus, the role of steroid hormones on endometriosis-associated ovarian cancers needs further study.

### 3.6. Small RNA Molecules

**Figure 2:**
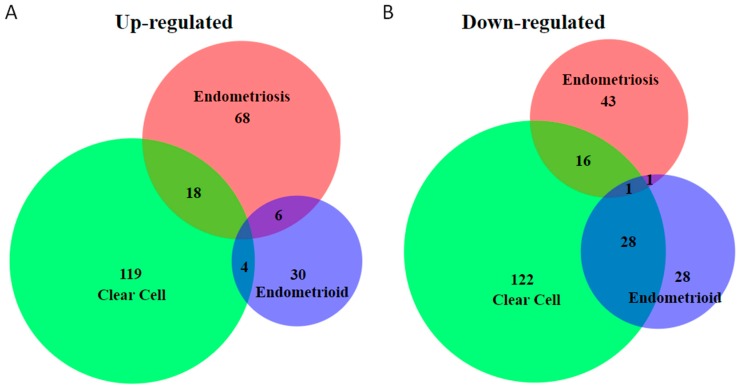
Venn diagram of overlap of number of miRNAs differentially expressed in endometriosis and ovarian clear-cell and endometrioid adenocarcinoma. The miRNAs differentially expressed are depicted in three overlapping circles. The numbers indicate the miRNA counts that are unique or in common between the groups. (**A**) Upregulated miRNAs; (**B**) downregulated miRNAs. Supplemental Table S1 details the miRNAs in each group above.

Small RNA molecules are non-coding RNA molecules that can play an important role in the post-transcriptional regulation of gene expression. Multiple groups of small RNAs have been identified, such as microRNAs (miRNAs), small nucleolar RNA (snoRNAs), small interfering RNAs (siRNAs), and Piwi-interacting RNA (piRNAs) [121]. The most studied type of small RNA molecules in endometriosis-associated ovarian cancers are miRNAs. In general, miRNAs regulate gene expression by mRNA cleavage and translational repression [122,123]. Studies have shown that miRNAs are frequently dysregulated in endometriosis and endometriosis-associated ovarian cancers (reviewed in [53,54,55]). Compilation of dysregulated miRNAs in ovarian endometrioid and clear-cell adenocarcinomas, as well as endometriosis (Supplemental Table S1) shows dysregulated miRNA molecules for each tissue type [53,55,124,125,126,127,128,129,130,131,132,133]. Figure 2 shows the number of miRNAs dysregulated in ovarian clear-cell and endometrioid adenocarcinomas, and endometriosis tissues. Supplemental Table S1 details the specific miRNA molecules in the each unique and overlapping group. MiR-126 was found downregulated in all three groups. While the function of miR-126 is still unknown, miR-126 was significantly downregulated in endometriosis compared with eutopic endometrium [134]. Additionally, downregulation of miR-126 induced non-ovarian cancer cell proliferation, migration, and invasion, mediated through numerous validated targets, such as PI3K, KRAS, and VEGF. Reduced levels of miR-126 were a significant predictor of poor survival of cancer patients, although women with ovarian cancer were not included in the study [135]. Thus, miR-126 may play a role in endometriosis and ovarian cancer, even though these functional studies did not have ovarian cancer samples with concurrent endometriosis.

MiR-30a, miR-30c, miR-31, miR-532-5p, and miR-885-5p were upregulated in clear cell ovarian cancer by multiple studies [124,125,126,127,131,132]. MiR-30 was found to be 5-fold overexpressed in ovarian clear-cell adenocarcinoma [132]. Sestito et al. showed that overexpression of miR-30a delayed tumor formation in xenograft tumors, and overexpression of miR-30a sensitized ovarian cancer cells to chemotherapy [136]. Downregulation of miR-532 was associated with poor survival in women with ovarian cancer, and overexpression of miR-532 suppressed the proliferative and invasive capacity of the ovarian cancer cell lines, ES2 and SKOV-3, and inhibited tumor growth in vivo [137]. Endometrioid ovarian cancer had the shortest list of dysregulated miRNAs (Figure 2 and Supplemental Table S1). MiR-200 family miRNAs (miR-200a, -200b, -200c, -141, and -429) were upregulated in ovarian cancer and may play crucial roles in ovarian cancer metastasis, diagnosis, and treatment [126,129,130,138].

## 4. Model Systems for Studying Rare Ovarian Cancers

Multiple model systems have been employed to study endometriosis and endometriosis-associated ovarian cancers (reviewed in [139,140]). This review will highlight the tumor microenvironment of the genetically engineered mouse models of endometriosis-associated ovarian cancers. We have chosen to focus on spontaneous models instead of transplant models (reviewed in [140]). Because there has yet to be a comprehensive mouse model that replicates ovarian cancer with endometriosis, this review will also focus on the role of immortalized cell lines, xenograft models, co-culture systems, and three-dimensional (3D) models.

### 4.1. Genetically Engineered Mouse Models

#### 4.1.1. Candidate Genes in Genetically Engineered Mouse Models

High-grade serous ovarian cancer is a genomically complex disease [141] and although neither endometrioid nor clear-cell ovarian cancer have been as extensively profiled, they are likely complex as well. For the study of genetically engineered mouse models, fortunately, both endometrioid and clear cell ovarian cancer have high frequency mutations in only a handful of genes: *ARID1A*, *PIK3CA*, *CTNNB1*, *PTEN*, and *KRAS* [33,40,41,42,44,45]. Use of traditional *Cre* recombinase technology with candidate-gene floxed alleles has had mixed results in terms of single gene knockout developing endometriosis-associated ovarian cancers. Table 1 lists the promoters driving *Cre* recombinase, and Table 2 details the brief rationale behind the use of specific genes in these mouse models. Table 3 lists these genes with combinations of tissue-specific promoters driving *Cre* recombinase. Despite the promising allele targets and the tissue-specific promoters driving *Cre* recombinase, there are no genetic mouse models of endometriosis and concurrent ovarian cancer. Investigators have created genetically engineered mouse models, which developed ovarian low-grade serous, clear-cell, or endometrioid adenocarcinoma (Table 3). However, none of these models have concurrent endometriosis. This suggests that different genetic combinations are required to model concurrent endometriosis and ovarian cancer. The discussion below highlights the role of the microenvironment of each model, and how this microenvironment may be playing a role in ovarian cancer development. Even though the presented models do not completely represent the endometriotic tumor microenvironment, they are still useful for understanding development of endometrioid or clear-cell ovarian cancer.

#### 4.1.2. Endometriosis

The only genetically engineered mouse model to spontaneously develop endometriosis with a single gene change is a highly innovative mouse model developed by Dinulescu et al. [162]. Using an oncogenic *KRAS* knock-in allele mouse (*Kras^G12D^*), peritoneal endometriosis developed after injection of adenovirus-driven *Cre* (Ad*^Cre^*) through the uterotubal junction to infect the ovarian bursa. This true peritoneal endometriosis model contained glandular epithelium and stromal components validated by molecular immunohistochemistry to cytokeratin 7, 8, and 20, estrogen receptor, progesterone receptor, smooth muscle actin, and CD10 [162]. Conversely, when Ad*^Cre^* was injected through the infundibulum to the ovarian bursa, the model develops ovarian endometriosis-like lesions without the stromal component [162]. A transplantation experiment hints that the peritoneal endometriosis is uterine or tubal in origin while the ovarian endometriosis-like lesions are ovarian surface epithelium derived [162]. While long-term follow up showed no development of ovarian cancer, future studies into the molecular lineage using secretory or ciliary markers may allow better definition of cell of origin [26,62]. A similar mouse model adds human mucin 1 (*MUC1*) to oncogenic *Kras^G12D^* with Ad*^Cre^* intrabursal injection [168]. This mouse model similarly exhibits endometriosis-like lesions of the ovary. Importantly, these mice developed an immune response to MUC1 with high numbers of CD4+ Foxp3+ regulatory T cells in para-aortic lymph nodes compared to uninjected mice without lesions [168]. Models which recapitulate the immune response are needed to study the endometriotic tumor microenvironment.

Because mice do not normally menstruate, modeling retrograde menstruation requires significant manipulation. In homologous mouse models of endometriosis, endometrium from an estrogen-primed donor mouse is injected into a syngeneic estrogen-treated recipient mouse. However, homologous mouse models such as these grow poorly without exogenous estrogen [162]. A variation is the menstrual mouse model. In this model, the donor mouse undergoes significant hormonal manipulation followed by a stimulation of the uterus leading to decidualization. Hormone withdrawal leads to degeneration of the endometrium with leukocyte invasion, similar to menstruation in women [169,170,171]. Donor sloughed endometrium is then placed into recipient syngeneic mouse. Using this approach, Cheng et al. placed oncogenic *Kras^G12V^* endometrial tissue into the subcuticular ventral abdomen of syngeneic mice without exogenous hormonal stimulation or matrix [172]. These lesions contained glandular epithelium, stroma, immune cells, extracellular matrix, and blood vessels with both estrogen receptor alpha and beta expression [172]. Similarly, Greaves et al. used a similar approach with endometrial tissue from a menstrual model of wild type mice. Using hormonally stimulated receptor mice, injection of tissue intraperitoneal with this non-genetically modified endometrial tissue leads to peritoneal endometriosis [173]. Again, these tissues were histologically and molecularly similar to human endometriosis [173]. Hormonal levels (i.e., endogenous versus exogenous high levels), tissue placement (i.e., subcuticular versus intraperitoneal), and genetic changes important to endometriosis-associated ovarian cancers (i.e., oncogenic *KRAS*, loss of function *ARID1A*) must be considered when using these menstrual endometriosis models. Additionally, genetically engineered mouse models that are unable to undergo decidualization such as *Pgr^Cre^;Arid1a^f/f^* mice [160] do not allow such studies.

#### 4.1.3. Clear Cell Ovarian Cancer

Poorly differentiated clear-cell ovarian carcinoma develops at 7.5 weeks post-injection in Ad^Cre^;Arid1a^f/f^;Pik3ca*^H1047R^ female mice with 77% penetrance and with 57% of injected mice having peritoneal metastasis [157]. Similar deletion of *ARID1A* alone or with knock-in of *Pik3ca* mutations showed ovarian surface epithelium hyperplasia but no endometriosis [157,158]. Although clear cell features are present two weeks post-injection, endometriotic-like lesions are not described [157]. Microarray analysis, comparing primary ovarian tumors to contralateral un-injected ovary, found almost 600 genes dysregulated with significant enrichment in immune system function [157]. Consistent with an endometriotic tumor microenvironment, IL6 signaling was found to be increased in the primary tumors, peritoneal metastases, body fluids, and ascites [157]. IL6 signaling and tumor cell growth was blocked with IL6 neutralizing antibodies. While IL6 expression was also implicated in normal ovarian surface epithelium hyperplasia with *ARID1A* deletion or *Pik3ca* mutation alone, the combination further enhanced IL6 production [157]. Cross-species, global gene expression profiling showed similar dysregulated genes in this mouse model compared to ovarian clear-cell adenocarcinoma from women [174]. Together these data suggest that the deletion of *ARID1A* and mutation in Pik3ca*^H1057R^ results in increased IL6 expression leading to the ovarian surface epithelial hyperplasia and eventually clear cell ovarian cancer. These tumor cells perpetuate IL6 production, creating a positive feedback loop of increased IL6 and increased cell (normal and cancerous) proliferation [157,174]. This interaction highlights how the tumor and its microenvironment can interact with one another to generate a more tumor-promoting environment.

#### 4.1.4. Endometrioid

*ARID1A*, *PIK3CA*, *CTNNB1*, *PTEN*, and *KRAS* [33,40,41,42,44,45] are commonly mutated in both endometrioid and clear cell ovarian cancers from women. However, manipulation of these genes in mice typically results in endometrioid but not clear cell ovarian cancer. On injection of adenovirus-driven Cre (*Ad^Cre^*) into the ovarian bursa through the infundibulum of *Pten^f/f^*;*Kras^G12D^* female mice generated female mice with 100% penetrance of highly aggressive and metastatic endometrioid ovarian cancer at 12 weeks. Interestingly, this mouse model has ovarian endometriosis-like lesions with either addition of oncogenic *Kras^G12D^* or deletion of *Pten* alone, but only results in endometrioid ovarian cancer when both *Pten* and *Kras^G12D^* are simultaneously mutated [162].

A mouse model targeting both *Pten* and *Apc* resulted in endometrioid ovarian cancer with high penetrance and metastatic disease [161]. Unfortunately, this conditional knockout (*Ad^Cre^*) did not result in endometriosis, which may be due to the early (6-week post-injection) tumor development [161]. Another model of endometrioid carcinoma in mice utilized a double conditional knockout of *Pten* and *Arid1a* and intrabursal *Ad^Cre^* injection to show a progression of ovarian surface epithelium hyperplasia, endometrioid carcinoma, and finally poorly differentiated carcinoma [158]. The well-differentiated endometrioid carcinoma was confined to the ovaries, suggesting the place of origin, while the undifferentiated tumors had metastasized into the peritoneal cavity [158]. Guan et al. hypothesizes that *ARID1A* plays a role in both tumor initiation and progression but requires the collaborative second hit of *Pten* to produce tumors [158]. Although the hyperplasia was not linked to endometriosis in these mice, it does speak to an environment of uncontrolled cellular proliferation giving rise to endometrioid ovarian cancer when left untreated.

High nuclear β-catenin levels have uniquely been found in endometrioid ovarian cancer from women, where this nuclear accumulation leads to activation of the WNT pathway [149]. Gain-of-function deletion of exon 3 of *Ctnnb1* leads to stable β-catenin expression in mice [175]. *Amhr2^Cre^Ctnnb1^f/f^* female mice have aggressive endometrioid ovarian cancers with 100% penetrance by 6 months. Addition of *Pten* deletion to this model allows for tumors that are even more aggressive by 6 weeks [165]. Similar to deletion of exon 3 of *Ctnnb1*, deletion of *Apc* leads to stable β-catenin and WNT signaling activation [149]. Only with deletion of *Pten* did mice develop ovarian tumors [161]. To model the progression of type I tumors to the more aggressive type II tumors, Wu et al. (2013) added *Pik3ca^E545K/+^* to *Apc^f/f^ Pten^f/f^* mice with Ad*^Cre^* and showed peritoneal and lung metastasis [167].

While these models used *Amhr2^Cre^* or Ad*^Cre^* to focus genetic changes in the ovarian surface epithelium, other studies have created conditional genetic changes in the oviduct. When *Apc* and *Pten* were concurrently deleted in the fallopian tube using *Ovgp1^Cre^*, endometrioid tumors of the ovaries developed in 10 of 15 mice, with 50% of those resulting in metastasis to the lungs or omentum [146]. Deletion of *Pten* in the fallopian tube by *Pax8^Cre^* also resulted in endometrioid tumors. Specifically, 75% of female mice developed primary tumors in the fallopian tube by 7 months, and 75% of tumor-burdened mice had metastasis to the ovaries [147]. Deletion of *Apc* with *Pgr^Cre^* female mice revealed tumors in both the oviduct and ovaries. Specifically, 25 of 40 female mice developed endometrioid oviductal tumors, one of 43 developed granulosa cell tumors, and 12 of 43 developed endometrioid ovarian tumors. While these female mice had simple ovarian cysts, the authors did not specifically denominate them as endometriosis [166]. Taken together, these mouse models suggest that the oviduct and/or the ovary may be involved in endometrioid cancer development in the mouse.

#### 4.1.5. Low-Grade Serous Ovarian Cancer

Addition of oncogenic *Kras* (*Kras^G12D^*) with either *Amhr2^Cre^* or *Cyp19^Cre^* resulted in ovaries with abnormal follicles, which were non-tumorigenic but also non-mitotic and non-apoptotic [145]. Deletion of *Pten* using *Amhr2^Cre^* did result in increased proliferation and increased cell survival of ovarian surface epithelium [163]. However, the loss of the tumor suppressor *Pten* alone is not tumorigenic in somatic cells of the ovary. When *Pten* is deleted in the context of oncogenic *Kras* with *Amhr2^Cre^*, there is development of low-grade serous papillary cystadenocarcinoma [163]. Although no endometriosis was noted, these mice were shown to have ovarian surface epithelium hyperplasia and abnormal follicle-derived ovarian lesions. Mullany et al. continued work on the *Kras^G12D^;Pten^f/f^;Amhr2^Cre^* mice and showed that ovarian surface epithelium cells, removed from mutant mice prior to tumor formation, developed into tumors when grown in soft agar [143]. This key result suggests that *Kras* and *Pte//* play a significant role in the development of tumors in the ovarian surface epithelium, and the genetic mutations are the primary driver, since tumor formation occurred even outside of the ovarian microenvironment [143].

### 4.2. Other Models

#### 4.2.1. Immortalized Cell Lines

Immortalized human ovarian cancer cell lines have been widely used for studying molecular mechanisms of ovarian cancer. Ovarian cancer cell lines are used to study cancer biology, connecting genetic and epigenetic alterations to cancer development, progression, and drug response. Importantly, ovarian cancer cell lines have been developed from different histological and molecular subtypes of ovarian cancer. Unfortunately, molecular characterization has revealed that common ovarian cancer cell lines (i.e., SKOV3, HEYA8) do not molecularly represent the histology of tumor of origin. The number of cell lines derived from either endometrioid or clear cell ovarian cancers is more limited than high-grade serous cell lines. However, molecular profiling, including attention to gene mutations common in these endometriosis-associated ovarian cancers (i.e., *ARID1A*, *PIK3CA*, *CTNNB1*, *PTEN*, and *KRAS*) and mutations common in high-grade serous (i.e., TP53), have allowed better molecular and biological distinction [176,177,178,179,180,181,182]. Table 4 shows the common endometrioid and clear-cell ovarian cancer cell lines, including lines that were not derived from endometriosis-associated ovarian cancers, but which may molecularly represent non-high grade serous cell lines. Even fewer cell endometriotic cell lines exist, with 12Z cells being the only widely shared epithelial-like endometriosis immortalized cell line [183]. For rigor and reproducibility, additional well-characterized endometriotic cell lines and possibly ovarian cancer cell lines derived from women with endometriosis need to be created.

#### 4.2.2. Xenograft Models

Implantation of immortalized human cell lines typically requires immunocompromised mice. A Japanese group created telomerase transformed endometriosis epithelial cell lines and confirmed cellular growth, steroid hormone response, and lack of malignant transformation in nude mice [185]. Further, these cells have been used in xenograft models to study treatment effects of small molecular inhibitors in endometriosis [104,200]. However, limited distribution outside Japan has restricted the use of these cells for studies of endometriosis-associated ovarian cancers. A similarly developed endometriotic epithelial cell line (EEC16) does not grow in SCID mice [184].

In terms of the endometriotic tumor microenvironment, Komiyama et al. placed normal endometrium of women without endometriosis into SCID mice. RMG-1 cells, a clear-cell ovarian cancer cell line, were grown in mice then transplanted into mice with or without endometrial implants. Although the tumors weighed less when grown with endometrium, proliferation was significantly higher in mice with transplanted endometrium. Additionally, these tumors expressed high levels of TGFβ and IL6. Addition of normal human endometrium changed the xenograft model to a more endometriotic microenvironment [201].

#### 4.2.3. Three Dimensional (3D) and Co-Culture Models

Immortalized cell lines in monolayer two-dimensional (2D) culture fail to recapitulate the complexity of tumor tissue. Tumors are three-dimensional (3D) structures, surrounded by other cell types and a unique extracellular matrix (ECM) that is biologically optimized for growth of each cell type [202]. To recapitulate this for in vitro model systems, immortalized cell lines can be grown in Matrigel, ultra-low-adhesive plates, or a hanging drop. Using these methods, many immortalized cell lines will form 3D spheroids. Three-dimensional spheroid models can be highly instructive towards the understanding of current drug resistance and new therapeutics because they better mimic the way 3D tumors or de novo spheroids interact with the surrounding microenvironment. Specifically, the architecture of spheroids results in non-heterogeneity of nutrient and drug penetration, which can cause differential responses to varying layers of the spheroid. For example, Lee et al. compared 31 ovarian cancer cell lines in both 2D monolayer and 3D spheroids to primary tumors. Three-dimensional spheroids showed slower rates of proliferation and decreased drug sensitivity than the same cells grown in 2D [202]. Additionally, these 3D spheroids mimicked histological characteristics of primary tumors. Although the authors did not perform genome-wide transcriptomic analysis, candidate biomarkers such as mucin 16, cell surface associated (CA125), Wilms Tumor 1 (WT1), estrogen receptor, Paired box gene 8 (PAX8), and β-catenin were examined by IHC on a tissue microarray composed of 2D and 3D samples. The expression of these biomarkers correlated well with expression in primary tumors [202]. These data suggest that 3D spheroid models alter the microenvironment in a potentially more biological way compared to other in vitro systems. Additionally, Lal-Nag et al. used high-throughput screening to test multiple oncological drugs against the HEYA8 cell line. The cells responded differently to various drugs if they were grown in monolayer, in the process of forming spheroids, or already in pre-formed spheroids. This work establishes that the dimensionality of ovarian cancer cells plays a role in how they respond to their environment [203]. Similarly, Chowwanadisai et al. created cisplatin-resistant ovarian cancer spheroids by treating cells with sub-threshold doses of cisplatin, which resulted in a mesenchymal-enriched gene expression signature [204]. While molecular changes within spheroids may play a role in chemotherapy resistance, size of spheroids, similar to remaining disease after debulking surgery, plays a role in response. Tanenbaum et al. [205] showed that small spheroids treated with either short-term high-dose or prolonged low-dose cisplatin underwent significant shrinkage. Importantly, large spheroids preferentially responded to short-term high doses of cisplatin [205]. The investigators did not explore if the remaining cells became chemotherapy-resistant [205]. Although immortalized cell lines from endometrioid or clear cell ovarian cancers have not been extensively tested in 3D culture, we anticipate that they would behave similarly.

In addition to single cell types within 3D spheroids, co-culture systems can be useful. For example, endometrial epithelial cells are inhibited at a rate of 65–80% when grown in co-culture with endometrial stromal cells, highlighting the need for complex co-culture models [206]. Additionally, co-culture models of epithelial and stromal endometriosis cells show that stromal cells are responsible for metabolism of iron. The authors hypothesize that storage of iron by stromal cells is protective against malignant transformation of epithelial cells. Specifically, a lack of stromal cells and an abundance of epithelial cells, which cannot metabolize iron, leads to oxidative damage and oncogenic change [207]. This hypothesis fits with data from Anglesio et al. showing tumorigenic mutations in *KRAS* in epithelial cells of endometriosis but not stromal cells [27]. Similarly, co-culture of macrophages with endometriotic epithelial or endometriotic stromal cells leads to an increase in invasion that is more robust in epithelial than stromal cells [208]. Three-dimensional organoids made from endometrium and decidua have been developed simultaneously by two independent laboratories and represent promising models for in vitro study [209,210]. Development of additional endometriotic tumor microenvironment models are needed to study ovarian cancer cells within spheroids, 3D organoids, or co-culture systems.

## 5. Future of Precision Therapy for/or Prevention of Ovarian Cancer

Endometriosis is a known risk factor for ovarian cancer [41]. However, early treatment of endometriosis represents a known prevention strategy for ovarian cancer. For example, a woman on oral contraceptive therapy has a more robust protection against ovarian cancer if she has endometriosis than if she does not [120]. While treatment of endometriosis with contraceptives is effective, women desiring fertility do not enjoy the side of effects of contraception, and when medical management is stopped, 73% of women have return of symptoms. Additionally, surgical treatment of endometriosis with removal of one or both ovaries results in significant decrease in ovarian cancer risk. However, 55% of women undergoing local resection of endometriosis will have at least one more surgery over the course of seven years [211,212]. Morbidity associated with multiple operations makes selection of timing for endometriosis surgery important in pre-menopausal women. New treatments for endometriosis are needed. Importantly, discovery of new treatments for endometriosis should be a priority for ovarian cancer funding agencies as these therapies may lead to prevention of ovarian cancer.

In terms of therapy highlighting the importance of the molecular signaling between cells within tumors, Mok et al. used a systems biology approach to study individual cell types. Machine learning with large databases of drugs and molecular effects highlighted an FDA-approved drug for potential targeted treatment. While this study used high-grade serous ovarian tumors, it brings forward the importance of non-epithelial ovarian cancer cells in cancer treatment [213]. Importantly, the study focused on TGFβ signaling pathways [213]. Endometriosis also has dysregulated TGFβ signaling pathways [92]. Similar treatment of endometriosis may prevent ovarian cancer.

## 6. Conclusions

The Gynecologic Cancers Steering Committee of the National Institutes of Health (NIH) proposed strategic priorities for ovarian cancer. These research priorities focus on discovery of biomarkers, identification of cancer subsets to drive treatment recommendations, immunotherapy, combination therapies, and manipulation of the host-tumor microenvironment. While these priorities are not specific for a particular histotype, they are highly applicable to both the more common high-grade serous and less common endometriosis-associated ovarian cancers and warrant further study in endometriosis-associated ovarian cancer models.

## Figures and Tables

**Table 1:** *Cre* recombinase promoters and site of effects.

*Cre*	Gene Promoter	Location of Expression	Ref.
Adenovirus (Ad)	Cytomegalovirus	Injection site	[142]
*Amhr2*	Anti-Mullërian hormone receptor type 2	Oviduct: stroma Uterus: stroma and smooth muscle cells Ovary: granulosa cells and ovarian surface epithelium	[143,144]
*Cyp19*	Cytochrome P450 family 19	Granulosa cells of antral follicles and luteal cells	[145]
*Ovgp1*	Oviductal glycoprotein 1	Non-ciliated oviductal epithelial cells	[146]
*Pax8*	Paired box gene 8	Fallopian tube, cervix, uterus, and endometrium	[147]
*Pgr*	Progesterone receptor	Oviduct: epithelium Uterus: epithelium, stroma, myometrium Ovary: time-limited granulosa cells	[148]

**Table 2:** Genes important in mouse models of endometriosis-associated ovarian cancer.

Mouse Allele	Gene Name and Mouse Ref	Effect of *Cre* Recombination	Endometriosis-Associated Ovarian Cancer Implications and Ref.
*Arid1a^f/f^*	AT-rich interactive domain 1A	ARID1A loss	46–95% of clear-cell and 30% of endometrioid tumors have loss of ARID1A [30,43,44,45]
*Apc^f/f^*	Adenomatous polyposis coli	Overexpression of β-catenin	Mutations in *APC* lead to activation of β-catenin which is frequently activated in endometrioisis-associated ovarian cancers [149]
*Ctnnb1^f/f^*	Catenin beta-1	Overexpression of β-catenin	16–54% of endometriod ovarian cancers have mutations in β-catenin, leading to nuclear localization, and activation of wingless integration site (WNT) signaling [150,151,152,153]
*Kras^lsl-G12D^*	Kirsten rat sarcoma	Expression of oncogenic *Kras*	29% of low-grade endometrioid ovarian tumors with concurrent endometriosis [33]
*MUC1^+/−^*	Mucin 1	Expression human MUC1 in mouse	Expressed in endometrium and endometriosis; potential biomarker for endometriosis or ovarian cancer [154]
*Pik3ca^H1047R^*	Phosphatidylinositol-4,5-bisphosphate 3-kinase catalytic subunit alpha	Mutation in *Pik3ca* kinase domain	20% of clear-cell and 20% of endometrioid ovarian cancers with mutations [155]
*Pten^f/f^*	Phosphatase and tensin homolog	PTEN loss and activation of AKT	20% of clear-cell and 20% of endometrioid cancers [156]

**Table 3:** Mouse models with implications in endometriosis and endometriosis-associated ovarian cancers.

Genotype	Phenotype	Penetrance	Details	Ref.
*Arid1a^f/f^;Ad^Cre^* (Ovarian bursa)	No cancer	0/29 with adnexal masses 0/42 with adnexal masses	No endometriosis	[157,158]
*Arid1a^f/f^;Amhr2^Cre^*	No cancer	0/20 with adnexal masses	No endometriosis	[159]
*Arid1a^f/f^;Pgr^Cre^*	No cancer	0/20 with adnexal masses	No endometriosis	[160]
*Pten^f/f^;Ad^Cre^* (Ovarian bursa)	No cancer	0/5 with adnexal masses 0/63 with adnexal masses	No endometriosis	[158,161]
*Pten^f/f^;Ad^Cre^* (Infundibulum to ovarian bursa)	Low penetrance endometrioid ovarian cancer at 26 weeks	8/13 with ovarian endometriosis like lesions 1/13 with ovarian cancer by 26 weeks	Endometriosis-like lesions of ovary (lacked stromal component)	[162]
*Pten^f/f^;Cyp19^Cre^*	No cancer	0/4 with adnexal masses	No endometriosis	[163]
*Pten^f/f^;Amhr2^Cre^*	Granulosa cell tumor	5/70 with ovarian cancers by 7 months	No endometriosis	[164]
*Pten^f/f^;Apc^f/f^;Ovgp1^Cre^*	Endometrioid ovarian carcinoma	10/15 with ovarian cancers	Metastatic lesions	[146]
*Pten^f/f^;* *Pax8^Cre^*	Endometrioid oviductal adenocarcinoma	3/4 with oviductal cancers by 7 months	Oviductal tumors metastasized to ovary	[147]
*Pik3ca^H1047R^;Ad^Cre^* (Ovarian bursa)	No cancer	0/6 with adnexal masses	4/5 ovarian surface epithelium hyperplasia (microscopic)	[157]
*Kras^G12D^;Ad^Cre^* (Infundibulum to ovarian bursa)	15/15 endometriosis-like lesions of ovary	15/15 with endometriosis-like lesions of ovary	Endometriosis-like lesions of ovary (lacked stromal component)	[162]
*Kras^G12D^;Ad^Cre^* (Uterotubal injection to ovarian bursa)	7/15 with peritoneal endometriosis	7/15 with peritoneal endometriosis	Peritoneal endometriosis	[162]
*Kras^G12D^;Ad^Cre^* (IP injection)	No cancer	0/13 with adnexal masses	No endometriosis	[162]
*Kras^G12D^;* *Amhr2^Cre^*	No cancer	0/4 with adnexal masses	No endometriosis Abnormal follicles	[145,163]
*Kras^G12D^;* *Cyp19^Cre^*	No cancer	0/4 with adnexal masses	No endometriosis Abnormal follicles	[145,163]
*Kras^G12D^;* *Pgr^Cre^*	No cancer	0/3 with adnexal masses	No endometriosis	[163]
*Ctnnb1^f/+^;Amhr2^Cre^*	Endometrioid ovarian carcinoma	5/6 with ovarian cancer by 6 months	No endometriosis	[165]
*Arid1a^f/f^;Pik3ca^H1047R^;Ad^Cre^* (Ovarian bursa)	Poorly differentiated clear-cell ovarian carcinoma	23/30 with ovarian cancer by 7 weeks	77% penetrance No endometriosis Aggressive metastatic tumors	[157]
*Arid1a^f/f^;Pten^f/f^;Ad^Cre^* (Ovarian bursa)	5/13 endometrioid ovarian carcinoma 8/13 undifferentiated adenocarcinoma	13/22 with ovarian cancer by 9 months	59% penetrance No endometriosis Aggressive undifferentiated tumors	[158]
*Apc^f/f^;Pgr^Cre^*	Endometrioid ovarian carcinoma	12/43 with ovarian cancer	No endometriosis 16% endometrioid ovarian cysts	[166]
*Pten^f/f^;Apc^f/f^;Ad^Cre^* (Ovarian bursa)	Endometrioid ovarian carcinoma	29/29 with ovarian cancer	100% penetrance No endometriosis Aggressive metastatic tumors	[161]
*Pten^f/f^;Apc^f/f^;Pik3ca^H1047R^;Ad^Cre^* (Ovarian bursa)	Endometrioid ovarian carcinoma	11/11 with ovarian cancer	No endometriosis Aggressive metastatic tumors	[167]
*Kras^G12D^;Pten^f/f^;Ad^Cre^* (Infundibulum to ovarian bursa)	Endometrioid ovarian carcinoma	9/9 with ovarian cancer by 12 weeks	100% penetrance Aggressive metastatic disease No endometriosis	[162]
*MUC1^+/-^;Kras^G12D^;Ad^Cre^* (Ovarian bursa)	Endometriosis-like lesions of ovary	No ovarian cancer	endometriosis-like lesions of ovary	[168]
*Ctnnb1^f/+^;Pten^f/f^;Amhr2^Cre^*	Endometrioid ovarian carcinoma	5/5 with ovarian cancer by 6 weeks	No endometriosis	[165]
*Kras^G12D^;* *Pten^f/f^;* *Amhr2^Cre^*	Low grade ovarian serous papillary adenocarcinomas	100% with ovarian tumors by 10 weeks	No endometriosis	[143,163]
*Kras^G12D^;* *Pten^f/f^;* *Pgr^Cre^*	No cancer	0/3 with adnexal masses	No endometriosis	[163]
*Kras^G12D^;* *Pten^f/f^;* *Cyp19^Cre^*	No cancer	0/3 with adnexal masses	No endometriosis	[163]

**Table 4:** Endometriosis and endometriosis-associated ovarian cancer cell lines.

Cell Line	Original Derivation	Putative Histotype by Molecular Studies	Genetic Mutations	Genetic Gains	Ref.
11Z	Red peritoneal endometriotic lesion	Benign	Unknown	Unknown	[183]
12Z	Red peritoneal endometriotic lesion	Benign (epithelial-like)	Unknown	Unknown	[183]
EEC16	Benign endometriotic lesion (epithelial-like)	Benign	Unknown	Unknown	[184]
EMosis-CC/TERT	Benign endometriotic lesion (epithelial-like)	Benign	Unknown	Unknown	[185]
22B	Red peritoneal endometriotic lesion (Stromal/fibroblast-like)	Benign	Unknown	Unknown	[183]
Hs 832(C).T (CRL-7566)	Benign endometriotic ovarian cyst	Benign	Unknown	Unknown	ATCC
OVTOKO	Clear-cell (spleen metastasis)	Clear-cell	None	*ERRB2*, *HNF1B*, *MET*, *PPM1D*, *STAT3*, *TP53*, *YAP1*, *ZNF217*, *CDKN2A*, *CDKN2B*	[177,178,179,182]
OVMANA	Clear-cell (primary tumor)	Clear-cell	*BRCA2*, *PIK3CA*, *ARID1A*	*ARID1A*, *MET*, *PPM1D*, *TP53*, *ZNF217*	[178,179,182,186]
TOV21G	Clear-cell (primary tumor)	Clear-cell	*KRAS*, *PTEN*, *PIK3CA*, *CTNNB1*, *ARID1A*, *TPX2*		[178,179,180,181,182,187]
RMG-1	Clear-cell (ascites)	Clear-cell	*TP53* *	*ERBB2*	[178,179,182,188]
RMG-2	Clear-cell	Clear-cell	*PPP2R1A*, *ARID1A*	*ERBB2*, *HNF1B*, *MET*, *PIK3CA*, *PPM1D*, *STAT3*, *ZNF217*, *CDKN2A*, *CDKN2B*	[179]
OCC1	Clear-cell	Clear-cell			[189]
JHOC-5	Clear-cell (pelvic metastasis)	Clear-cell		*ARID1A*, *ERBB2*, *HNF1B*, *MET*, *PIK3CA*, *PPM1D*, *STAT2*, *ZNF217*, *CDKN2A*, *CDKN2B*	[178,179,182,190]
JHOC-7	Clear-cell	Clear-cell	*PIK3CA*	*ARID1A*, *HNF1B*, *PIK3CA*, *PPM1D*, *STAT3*, *ZNF217*	[179]
JHOC-9	Clear-cell	Clear-cell	*PTEN*, *ARID1A*	*HNF1B*, *ZNF217*	[179]
ES2	Poorly differentiated clear-cell (primary tumor)	Endometrioid/Clear-cell	*BRAF*, *TP53*, *APC*, *MYC*		[178,179,180,181,182,191]
OVISE	Clear-cell (pelvic metastasis)	Endometrioid/Clear-cell	*ARID1A*		[177,178,179,182]
OVSAYO	Clear-cell	Serous	*TP53*		[179]
TOV112D	Endometrioid (primary tumor)	Endometrioid	*CTNNB1*, *TP53*		[179,180,181,182,187]
OVK18	Endometrioid (ascites)	Endometrioid	*TP53*, *PTEN*, *KRAS*, *ARID1A*		[178,182,192]
SNU-251	Endometrioid	Endometrioid	*BRCA1*		[193]
2008	Endometrioid	Atypical non-serous	*TP53*		[179]
IGROV1	Endometrioid with serous/clear cell (primary tumor)	Endometrioid/Clear-cell	*PTEN*, *TP53*, *ARID1A*, *BRCA1*, *BRCA2*, *PIK3CA*, *TPX2*		[178,179,180,182,194]
59M	Endometrioid with clear cell (ascites)	Endometrioid/Clear-cell	*TP53*	*MYC*	[178,180,182,193,195]
COV362	Endometrioid (pleural effusion)	Serous	*TP53*, *BRCA1*, *RB1* *, *EGFR*, *APC*	*MYC*	[178,180,182,196]
A2780	Unknown adenocarcinoma	Endometrioid	*PTEN*, *ARID1A*, *PIK3CA*, *BRAF*		[178,179,180,181,182,197]
HEYA8	Moderately differentiated papillary serous (peritoneal metastasis)	Unlikely serous	*KRAS*, *BRAF*		[178,179,182,198]
SKOV3	Well differentiated, adenocarcinoma (ascites)	Endometrioid/Clear-cell	*PIK3CA*, *ARID1A*	*ERBB2*	[178,179,180,181,182,199]

* Homozygous deletion.

## Supplementary Materials

The following are available online at http://www.mdpi.com/2072-6694/10/8/261/s1, Table S1: Dysregulated miRNAs in ovarian endometrioid and clear-cell adenocarcinoma and endometriosis.
